# Immune landscape in rejection of renal transplantation revealed by high-throughput single-cell RNA sequencing

**DOI:** 10.3389/fcell.2023.1208566

**Published:** 2023-07-20

**Authors:** Ning Wen, Jihua Wu, Haibin Li, Jixiang Liao, Liugen Lan, Xiawei Yang, Guangyi Zhu, Zhiying Lei, Jianhui Dong, Xuyong Sun

**Affiliations:** ^1^ Transplant Medical Center, The Second Affiliated Hospital of Guangxi Medical University, Nanning, China; ^2^ Guangxi Key Laboratory of Organ Donation and Transplantation, Nanning, China; ^3^ Guangxi Clinical Research Center for Organ Transplantation, Nanning, China

**Keywords:** renal transplantation, rejection, single-cell RNA sequencing, immune landscape, pro-inflammatory response, oxidative stress

## Abstract

**Background:** The role of the cellular level in kidney transplant rejection is unclear, and single-cell RNA sequencing (scRNA-seq) can reveal the single-cell landscape behind rejection of human kidney allografts at the single-cell level.

**Methods:** High-quality transcriptomes were generated from scRNA-seq data from five human kidney transplantation biopsy cores. Cluster analysis was performed on the scRNA-seq data by known cell marker genes in order to identify different cell types. In addition, pathways, pseudotime developmental trajectories and transcriptional regulatory networks involved in different cell subpopulations were explored. Next, we systematically analyzed the scoring of gene sets regarding single-cell expression profiles based on biological processes associated with oxidative stress.

**Results:** We obtained 81,139 single cells by scRNA-seq from kidney transplant tissue biopsies of three antibody-mediated rejection (ABMR) patients and two acute kidney injury (AKI) patients with non-rejection causes and identified 11 cell types, including immune cells, renal cells and several stromal cells. Immune cells such as macrophages showed inflammatory activation and antigen presentation and complement signaling, especially in rejection where some subpopulations of cells specifically expressed in rejection showed specific pro-inflammatory responses. In addition, patients with rejection are characterized by an increased number of fibroblasts, and further analysis of subpopulations of fibroblasts revealed their involvement in inflammatory and fibrosis-related pathways leading to increased renal rejection and fibrosis. Notably, the gene set score for response to oxidative stress was higher in patients with rejection.

**Conclusion:** Insight into histological differences in kidney transplant patients with or without rejection was gained by assessing differences in cellular levels at single-cell resolution. In conclusion, we applied scRNA-seq to rejection after renal transplantation to deconstruct its heterogeneity and identify new targets for personalized therapeutic approaches.

## 1 Introduction

Kidney transplantation is a very effective treatment for end-stage renal disease, but complications after kidney transplantation have been a major clinical problem and long-term survival rates have been suboptimal (Augustine, 2018). Rejection reactions remain a major threat and are the main independent risk factor for long-term survival of transplanted kidneys (Cooper, 2020; Wang et al., 2021). The two phases of immune response in allografts are different in early and late stages. In the past, graft acceptance without long-term immunosuppression was not uncommon in living transplants (Lim et al., 2017). Although modern immunosuppressive techniques are now available and the short-term results of kidney transplantation have improved to >90% within 1 year, the long-term results over 20 years have not improved significantly (Valenzuela and Reed, 2017; Eskandary et al., 2018). Fibrotic arterial thickening, interstitial fibrosis and tubular atrophy not only severely affect graft function, but also survival (Wekerle et al., 2017). A large amount of data has been established for bulk transcription analysis to predict graft outcome. However, these analyses are limited, and traditional bulk RNA-seq and kidney biopsy methods ignore transcriptome heterogeneity at single-cell resolution (Liao et al., 2020).

Single-cell RNA sequencing (scRNA-seq) plays a key role in identifying cellular subtypes and elucidating differential expression between genes (Saliba et al., 2014; Li et al., 2019; Zhao et al., 2020). Recently, there have been some new discoveries about scRNA-seq, revealing the mechanism of kidney disease (Wu et al., 2018). For example, scRNA-seq analysis of systemic lupus erythematosus nephritis identified high expression of interferon-inducible genes in renal tubular cells associated with disease severity (Der et al., 2017). Currently, our understanding of the single-cell level of rejection, especially antibody-mediated rejection (ABMR), is poor. Numerous analyses have shown that ABMR is the leading cause of late-stage allograft failure (Sellares et al., 2012). The balance of the inflammatory and anti-inflammatory responses of the immune system, and the cytokine storm caused by the immune response after transplantation may cause rejection, thereby affecting the survival rate of transplantation (Salehi et al., 2020). Therefore, analysis of cytokine production at the single cell level may be important to elucidate the fate of the graft.

In this study, we explored changes at the single-cell level in kidney transplanted living tissue from 5 patients with AKI (including 3 with ABMR and 2 nonrejection cause), which in turn determined the pro-inflammatory parenchymal response in the rejected kidney. Our data provide biological insights into renal transplant rejection by constructing a very comprehensive single-cell landscape of renal transplant rejection, describing the molecular function of the cells, which will aid reduce the adverse reactions after renal transplantation.

## 2 Materials and methods

### 2.1 Data collection and processing

The GPL24676 platform-based renal transplant biopsy-related scRNA-seq dataset GSE145927 (Malone et al., 2020) was downloaded from Gene Expression Omnibus (GEO, https://www.ncbi.nlm.nih.gov/geo/). A total of 5 patients with AKI (including 3 with ABMR and 2 nonrejection cause) were included and their kidney allograft tissue was biopsied, with biopsies performed between 5 days and 7 years after transplantation. All patients diagnosed with ABMR had donor-specific antibodies (DSA) at the time of biopsy (Supplementary Table S1). In addition, whole-exome sequencing data for donor and recipient DNA in the dataset were not consistent with this study, and this data was not included in the study.

### 2.2 Construction of single cell atlas

Quality control of single-cell Seurat objects on double cell numbers, dead cells, and mitochondrial genes using Seurat package (Stuart et al., 2019) in R language. For all downstream analyses, we selected cells that have at least 1,000 unique molecular identifiers (UMIs) (indicating the number of captured transcripts) mapped to at least 200 unique genes and ensured that each gene is expressed in more than 3 cells. We excluded cells with poor viability and quality by removing more than 10% of the cells whose gene counts reflected mitochondrial genes or ribosomal RNA (Supplementary Figure S1). Single-cell data were merged using the IntegrateData function (Butler et al., 2018) of the Seurat package (Stuart et al., 2019) in R language to perform cell clustering analysis according to default parameters. The clustering results were downscaled and visualized (Becht et al., 2018) based on the uniform manifold approximation and projection (UMAP) technique, and projected onto a two-dimensional image defined as a single-cell atlas. In addition, the FindAllMarkers function of the Seurat package was used to identify genes specifically expressed in each cell cluster, with *p*-value < 0.05 and |log fold change (logFC)| > 0.5 being considered significant. In addition, cell types were annotated according to cellular markers known from previous studies (Malone et al., 2020).

### 2.3 Differential gene expression analysis

At the single-cell level, differentially expressed genes in each cluster between allograft biopsies from patients with and without rejection were determined using the “FindAllMarkers” function, and differences associated with adjusted *p*-value < 0.05 were considered significant.

### 2.4 Functional enrichment and gene enrichment analysis

To explore the biological processes and pathways involved in the marker for each cell cluster, the R package clusterProfiler (Yu et al., 2012) was applied to the marker for enrichment analysis regarding the biological processes (BPs) of gene ontology (GO) and the Kyoto Encyclopedia of Genes and Genomes (KEGG) signaling pathway, with *p*-value < 0.05 BPs and KEGG signaling pathways were considered significant.

### 2.5 Pseudotime analysis

Based on the changes in gene expression of different cell subpopulations over time, we reconstructed the developmental trajectory of dysregulated cell differentiation in renal transplant rejection using the Monocle 3 package (Trapnell et al., 2014) in R language to explore the changes in dysregulated immune cells during the development of renal transplant rejection.

### 2.6 Gene regulatory network (GRN) analysis

In addition, using the Python module tool pySCENIC (Van de Sande et al., 2020), this study comprehensively reconstructed the transcription factor-centered gene regulatory network to further explore the regulatory mechanisms of dysregulated cells. The tool first uses a per-target regression approach (GRNBoost2) to infer co-expression modules, then uses cis-regulatory motif discovery (cisTarget) to prune indirect targets from these modules, and finally quantifies the activity of these regulators by enrichment scores of regulator target genes.

### 2.7 Cellular scoring of oxidative stress-related gene sets

The AddModuleScore function (Tirosh et al., 2016) in the seruat package was used to score the specified gene sets. Oxidative stress-related pathway dataset from the Gene Set Enrichment Analysis (GSEA) database (Supplementary Table S2). This function is to calculate module scores for feature expression programs in single cells in the oxidative stress-related pathway dataset.

#### 2.7.1 Data Analysis and Statistics

All statistical analyses were performed in R package. Comparisons between the two groups were made using Student’s t test and correlation coefficients were calculated using Spearman analysis. *p*-value < 0.05 was considered significant.

## 3 Results

### 3.1 Global single-cell landscape of renal transplant patients

**FIGURE 1:**
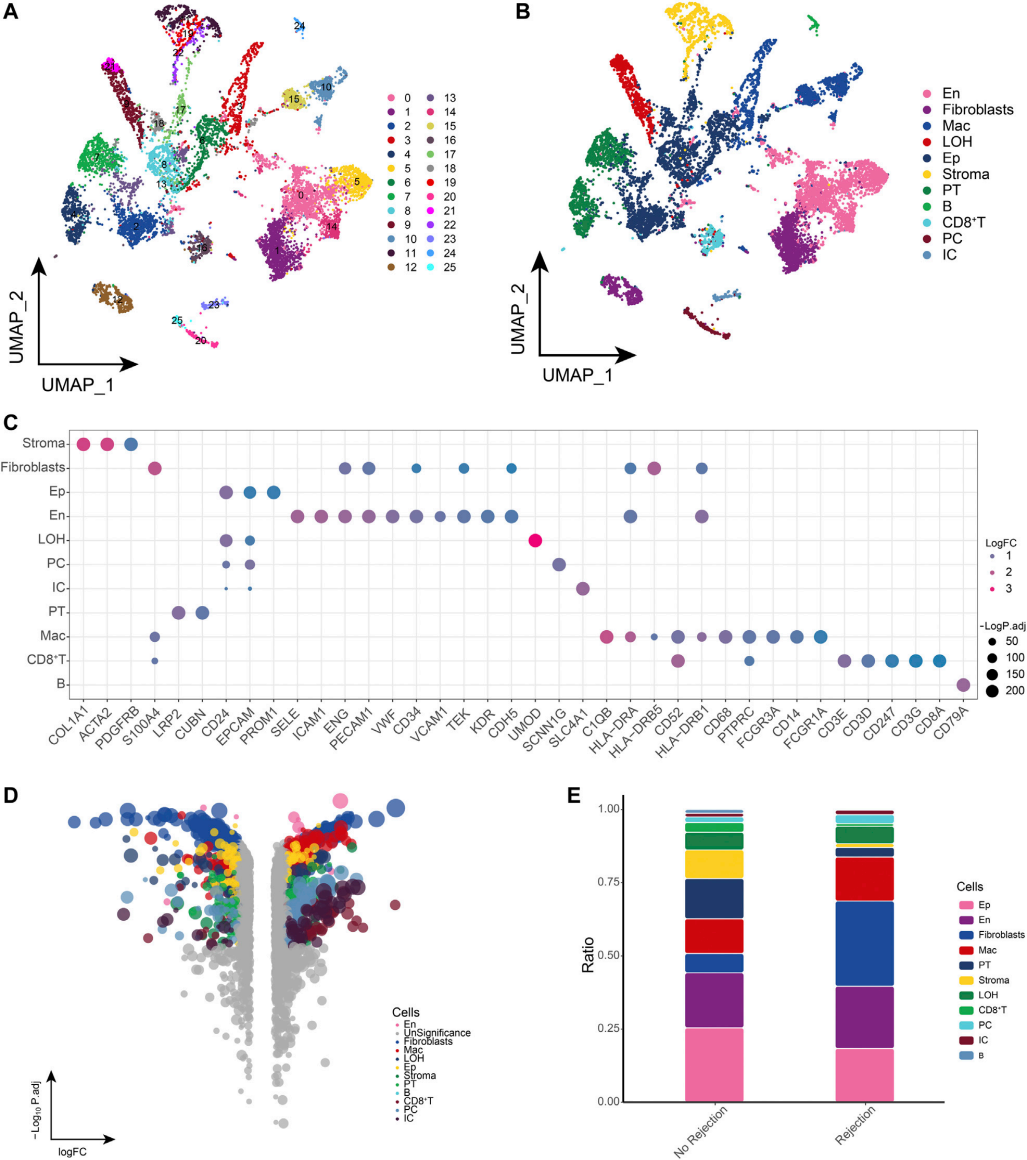
Global single-cell landscape of renal transplant patients **(A)**. Single-cell process based on a total of 26 cell clusters captured. **(B)** Single-cell atlas mapping cell types. **(C)** CellMarker-based bubble map showing global landscape of cell types. **(D)** Multiset volcano map showing differentially expressed genes for each cell type in patients with and without rejection kidney transplantation. **(E)** Differences in cell abundance between renal transplant patients and patients without rejection.

We obtained single-cell sequencing data from public databases of biopsied renal allograft tissue from three patients with ABMR and two patients with a nonrejection cause of AKI to further explore the potential ecological panoply of renal transplant patients. After standardized data processing and quality control, a total of 81,139 high quality single cells were obtained and clustered to generate 26 cell clusters (Figure 1A), including 11 cell types such as endothelial cells (En), epithelial cells (Ep), fibroblasts, macrophages (Mac), loop of henle (LOH), stromal cells (Stroma), proximal tubule (PT), principal cells (PC), B cells, CD8^+^ T cells, and intercalated cells (IC) (Figure 1B). The cell clusters positively expressed markers were consistent with the gene signatures published in recent scRNA-seq and laboratory studies, among others (Wu et al., 2018; Malone et al., 2020), in line with the phenotypic characteristics of the corresponding cells (Figure 1C). Differential gene expression analysis revealed a general dysregulation of gene expression among different cell types in the ABMR group relative to the nonrejection patient group (Figure 1D). Further comparison of the differences in cell composition between rejection-free, ABMR patients revealed a dramatic increase in fibroblast abundance in ABMR patients, while an increased abundance of macrophages was observed (Figure 1E). In summary, we initially constructed a dynamic single-cell ecological global landscape of the microenvironment of biopsied renal allograft tissue in renal transplant patients by single-cell histology, and we found significant gene expression dysregulation accompanying between different cell types and explored the altered cellular ecology in nonrejection and ABMR patients.

### 3.2 Landscape of fibroblast subpopulations in renal transplant patients

**FIGURE 2:**
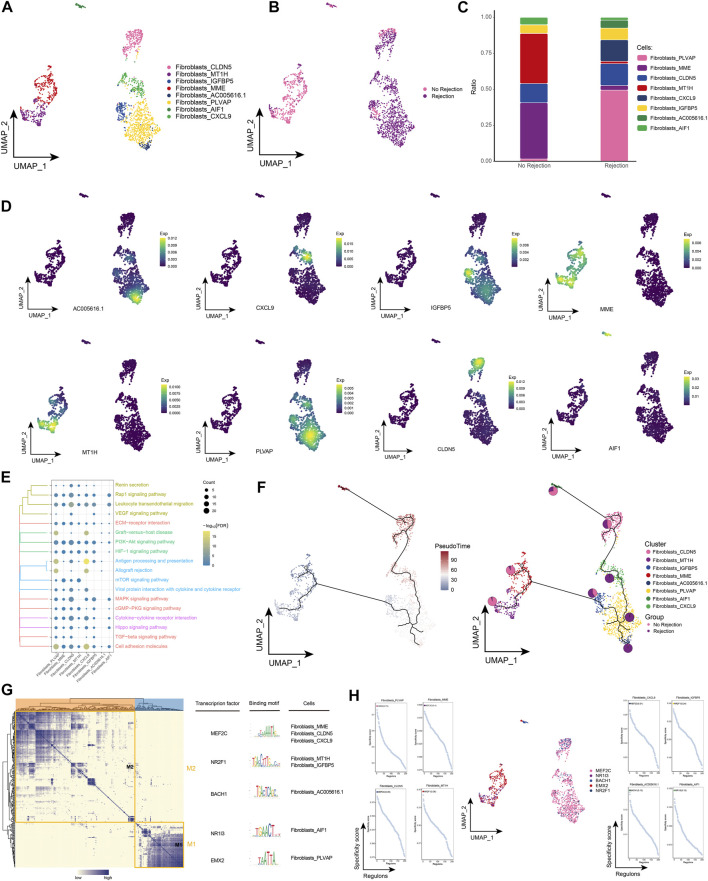
Fibroblast subpopulations in renal transplant patients **(A)**. Single-cell atlas demonstrating fibroblast subpopulations. **(B)** Single-cell atlas demonstrating fibroblast subpopulations in renal transplant patients with and without rejection. **(C)** Differences in abundance of fibroblast subpopulations in renal transplant patients with and without rejection. **(D)** Markers predominantly expressed in fibroblast subpopulations. **(E)** Biological processes and signaling pathways underlying the significantly increased enrichment of fibroblast subpopulations in the microenvironment of renal transplant patients. **(F)** Pseudotime developmental trajectory of fibroblast subpopulations, with pie charts representing the proportion of fibroblast subpopulations in patients with and without rejection kidney transplant. **(G)** Co-expression modules of transcription factors in fibroblast subpopulations of renal transplant patients. Left panel: identification of regulator modules based on the regulator’s linkage specificity index matrix. Middle: representative transcription factors and their binding patterns in the modules. Right panel: cell subpopulations in which transcription factors are located. **(H)** Single-cell atlas showing transcription factors regulating fibroblast subpopulations.

Fibroblasts are thought to be closely associated with allograft fibrosis (Wang et al., 2017), and in our study, we found significant fiber abundance in ABMR patients. Based on a single-cell resolution cellular ecological atlas, we further explored the subpopulations of fibroblasts in renal transplant patients and identified a total of eight subpopulations of fibroblasts (Figure 2A) and found that these subpopulations were heterogeneous among different subgroups (Figure 2B). Among these fibroblast subpopulations, we found that Fibroblasts_PLVAP and Fibroblasts_CXCL9 had the highest proportions in ABMR patients, and Fibroblasts_MME and Fibroblasts_MT1H had the highest proportions in the group of patients with a nonrejection cause (Figure 2C), while specific markers for these fibroblast subpopulations were mapped in the single cell atlas (Figure 2D). By enrichment analysis, these specifically altered fibroblast subpopulations were found to be significantly enriched for Allograft rejection, Graft-versus-host disease and Renin secretion, which are relevant pathways regarding post-transplant rejection, and in addition, we observed that these subpopulations are also involved in the TGF-β signaling pathway, epithelial to mesenchymal transition (EMT) and angiogenesis-related pathways (Figure 2E). These fibroblast subpopulations underwent a continuous developmental process, with Fibroblasts_PLVAP located at the beginning of development (Figure 2F). Subsequent GRN analysis showed that this subpopulation of genes was organized into two modules (Figure 2G), each subject to transcription factors (TFs) that regulate specific gene expression in kidney transplant patient-specific fibroblasts to guide cell fate selection (Figure 2H). These results reflect that the Fibroblasts_PLVAP and Fibroblasts_CXCL9 subpopulations were activated and associated with EMT of the renal tubules in patients with ABMR compared to patients with a nonrejection cause, which may have implications for progressive tubulointerstitial fibrosis.

### 3.3 Landscape of epithelial cell subpopulations in renal transplant patients

**FIGURE 3:**
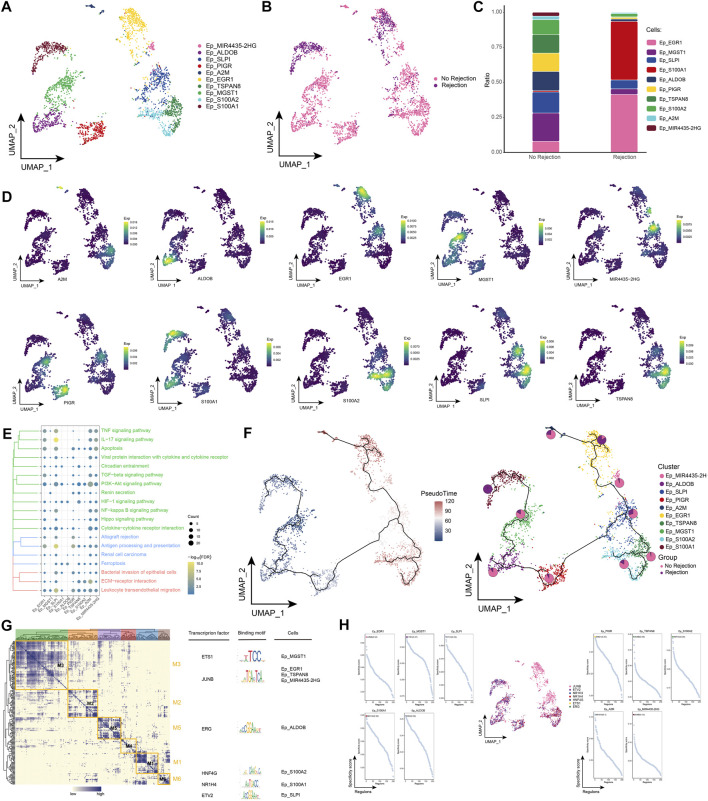
Epithelial cell subpopulations in renal transplant patients **(A)**. Single-cell atlas demonstrating into epithelial cell subpopulations. **(B)** Single-cell atlas demonstrating epithelial cell subpopulations in patients with and without rejection kidney transplantation. **(C)** Differences in abundance of epithelial cell subpopulations in patients with and without rejection kidney transplantation. **(D)** Markers predominantly expressed in epithelial cell subpopulations. **(E)** Biological processes and signaling pathways underlying the significantly increased enrichment of epithelial cell subpopulations in the microenvironment of renal transplant patients. **(F)** Pseudotime developmental trajectory of epithelial cell subpopulations with pie charts representing the proportion of epithelial cell subpopulations in rejection and rejection-free renal transplant patients. **(G)** Co-expression modules of transcription factors in epithelial cell subpopulations from renal transplant patients. Left: Identification of regulator modules based on the regulator’s linkage specificity index matrix. Middle: representative transcription factors and their binding patterns in the modules. Right panel: cell subpopulations in which transcription factors are located. **(H)** Single-cell atlas showing transcription factors regulating epithelial cell subpopulations.

Extensive immune system activation can cause necrosis of renal tubular epithelial cells, rupture of the basement membrane, and eventually fibrosis and loss of renal transplant function (Rogers et al., 2016). By subpopulation analysis, ten epithelial cell subpopulations were further identified in this study (Figure 3A) and the distribution of these subpopulations in different patient groups was observed (Figure 3B). Among them, Ep_S100A1 and Ep_EGR1 were highly represented in patients with ABMR compared to patients with a nonrejection cause (Figure 3C), and the expression of specific marker genes of these subpopulations was further observed at single-cell resolution (Figure 3D). Subsequently, enrichment analysis revealed that these specifically altered epithelial cell subpopulations were significantly enriched for signaling pathways associated with post-transplant rejection, such as Allograft rejection, Graft-versus-host disease, and Renin secretion, and were also involved in pan-death pathways, IL-17 signaling pathway, Cell adhesion molecules, Leukocyte transendothelial migration, and ECM-receptor interaction (Figure 3E). In addition, pseudotime analysis revealed high mimetic time values for Ep_S100A1 and Ep_EGR1, indicating that they are at the end of the developmental trajectory (Figure 3F). By GRN, we explored TFs that regulate epithelial cell subpopulations (Figures 3G, H).

### 3.4 Landscape of LOH cell subpopulations in renal transplant patients

**FIGURE 4:**
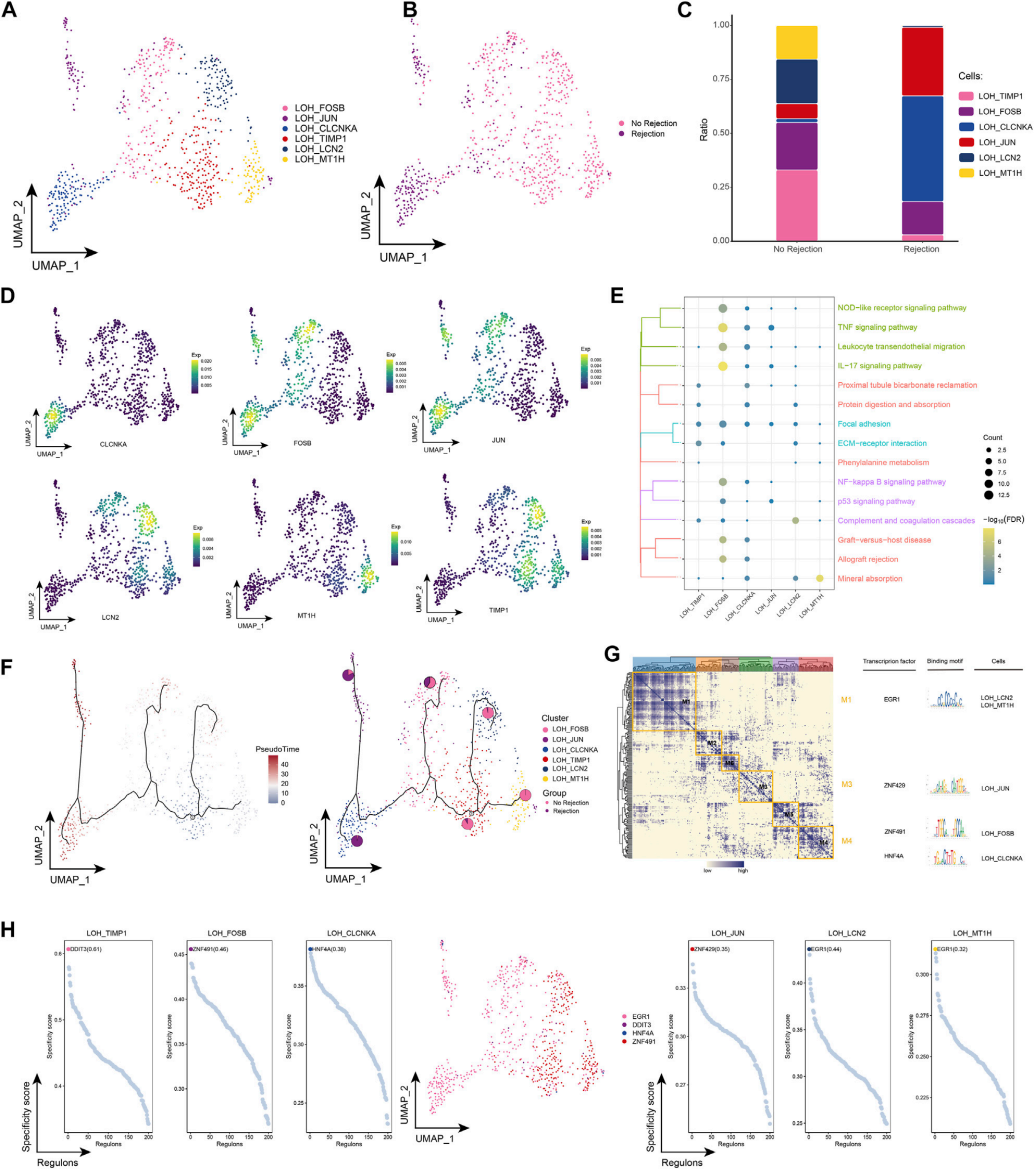
Renal transplant patients with LOH cell subpopulation **(A)**. Single-cell atlas demonstrating into LOH cell subpopulations. **(B)** Single-cell atlas demonstrating LOH cell subpopulations in renal transplant patients with and without rejection. **(C)** Differences in LOH cell subpopulation abundance in renal transplant patients with and without rejection. **(D)** Markers predominantly expressed in LOH cell subsets. **(E)** Biological processes and signaling pathways underlying the significantly increased enrichment of LOH cell subsets in the microenvironment of renal transplant patients. **(F)** Pseudotime developmental trajectory of LOH cell subsets, with pie charts representing the proportion of LOH cell subsets in patients with and without rejection kidney transplantation. **(G)** Co-expression modules of transcription factors in LOH cell subpopulations from renal transplant patients. Left panel: identification of regulator modules based on the regulator’s linkage specificity index matrix. Middle: representative transcription factors and their binding patterns in the modules. Right panel: cell subpopulations in which transcription factors are located. **(H)** Single-cell atlas showing transcription factors regulating LOH cell subpopulations.

By subpopulation analysis, we further identified six LOH cell subpopulations (Figures 4A, B). In addition, we found that LOH_CLCNKA and LOH_JUN were highly represented in patients with ABMR compared to patients with a nonrejection cause (Figure 4C) and further explored the expression of specific markers for these subpopulations by single-cell resolution (Figure 4D). Enrichment analysis revealed that cell subpopulations of LOH were significantly involved in signaling pathways related to post-transplant rejection such as Proximal tubule bicarbonate reclamation, Graft-versus-host disease and Allograft rejection, and also significantly involved in IL-17 signaling pathway and TNF signaling pathway of inflammation-related pathways (Figure 4E). By pseudotime analysis, LOH_CLCNKA and LOH_JUN were found to be at the end of the developmental trajectory of LOH cells (Figure 4F). In addition GRN showed that LOH cells were regulated by TFs such as EGR1, ZNF429, ZNF491, and HNF4A, respectively (Figures 4G, H). In conclusion, this study identified LOH cell subpopulations in renal transplant patients and found that these subpopulations were significantly involved in a number of signaling pathways related to post-transplant rejection and inflammation-related pathways. In addition, the transcriptional regulation of cell subpopulations during their differentiation was further elucidated by inferring the developmental trajectories of LOH cell subpopulations.

### 3.5 Landscape of macrophage subpopulations in renal transplant patients

**FIGURE 5:**
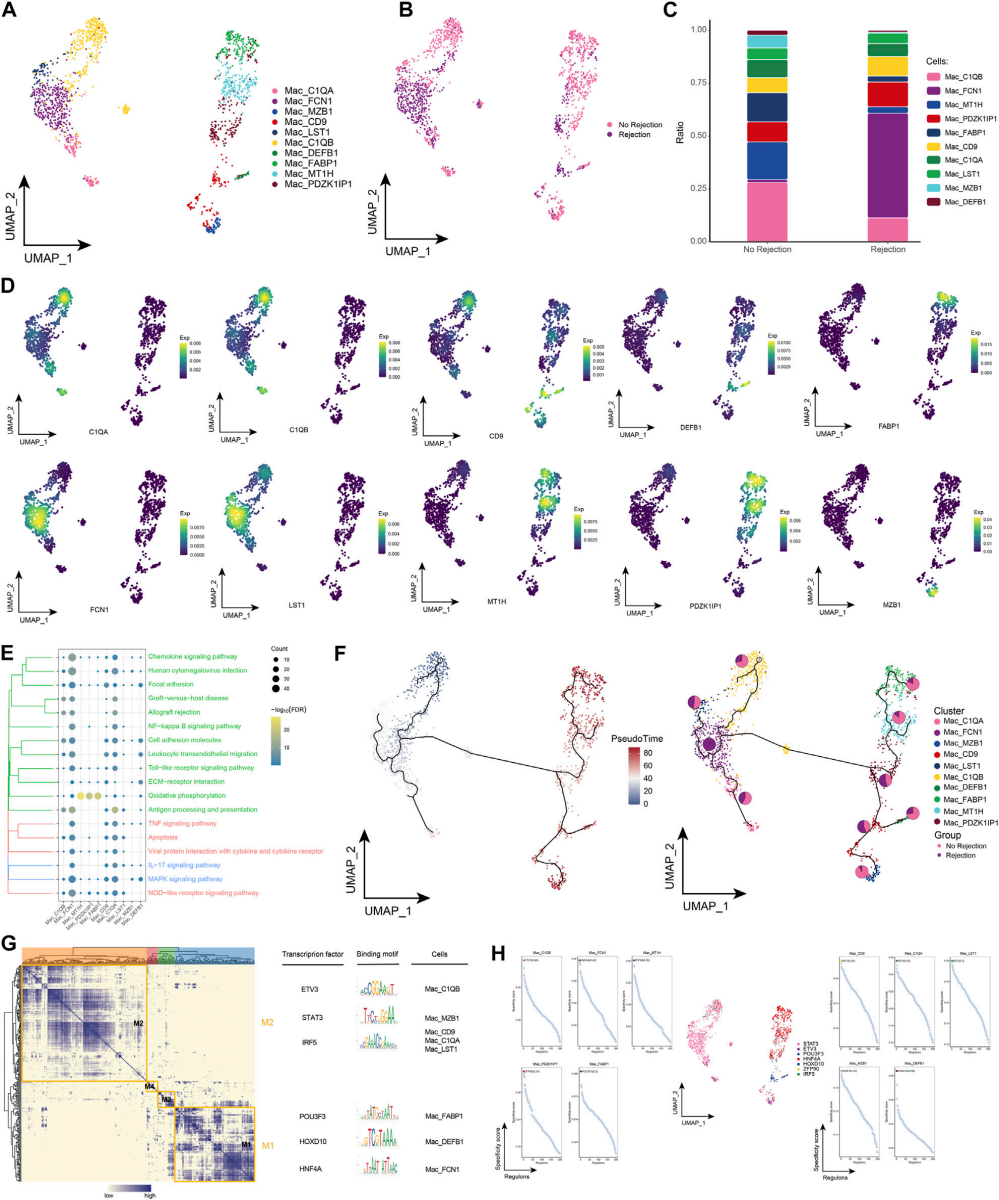
Macrophages subpopulations in renal transplant patients **(A)**. Single-cell atlas demonstrating into macrophages subpopulations. **(B)** Single-cell atlas demonstrating macrophages subpopulations in renal transplant patients with and without rejection. **(C)** Differences in macrophages subpopulation abundance in renal transplant patients with and without rejection. **(D)** *Major* markers expressed by macrophages subpopulations. **(E)** Biological processes and signaling pathways underlying the significantly increased enrichment of macrophages subpopulations in the microenvironment of renal transplant patients. **(F)** Pseudotime developmental trajectory of macrophages subpopulations, with pie charts representing the proportion of macrophages subpopulations in patients with and without rejection kidney transplantation. **(G)** Co-expression modules of transcription factors in macrophages subpopulations from renal transplant patients. Left panel: identification of regulator modules based on the regulator’s linkage specificity index matrix. Middle: representative transcription factors and their binding patterns in the modules. Right panel: cell subpopulations in which transcription factors are located. **(H)** Single-cell atlas showing transcription factors regulating macrophages subpopulations.

We also performed a more refined typing of macrophages in renal transplant patients. Ten macrophage subpopulations were identified by subpopulation analysis in this study, and again these subpopulations showed heterogeneity across patient groups (Figures 5A, B). Notably, we found the highest proportion of Mac_FCN1 in patients with ABMR compared to patients with a nonrejection cause (Figure 5C), and the specific marker of Mac_FCN1 was verified by single-cell resolution (Figure 5D). We then further explored the pathways involved in the macrophage subpopulation in kidney transplant patients and showed that this subpopulation was significantly enriched in Chemokine signaling pathway, TNF signaling pathway and Viral protein interaction with cytokine and cytokine receptor, in addition to some post-transplant rejection-related pathways were also enriched (Figure 5E). The pseudotime analysis showed that this subpopulation is early in the developmental trajectory of macrophages, indicating that this subpopulation also has a high developmental potential (Figure 5F). Subsequently, by GRN, we identified a series of TFs regulating the macrophage subpopulation (Figures 5G, H). In conclusion, by identifying macrophage subpopulations, we identified the Mac_FCN1 subpopulation, that is, highly represented in the microenvironment of patients with ABMR after renal transplantation, which has a high differentiation potential and may play an important role in the inflammatory response after renal transplantation.

### 3.6 Landscape of stromal cell subpopulations in renal transplant patients

**FIGURE 6:**
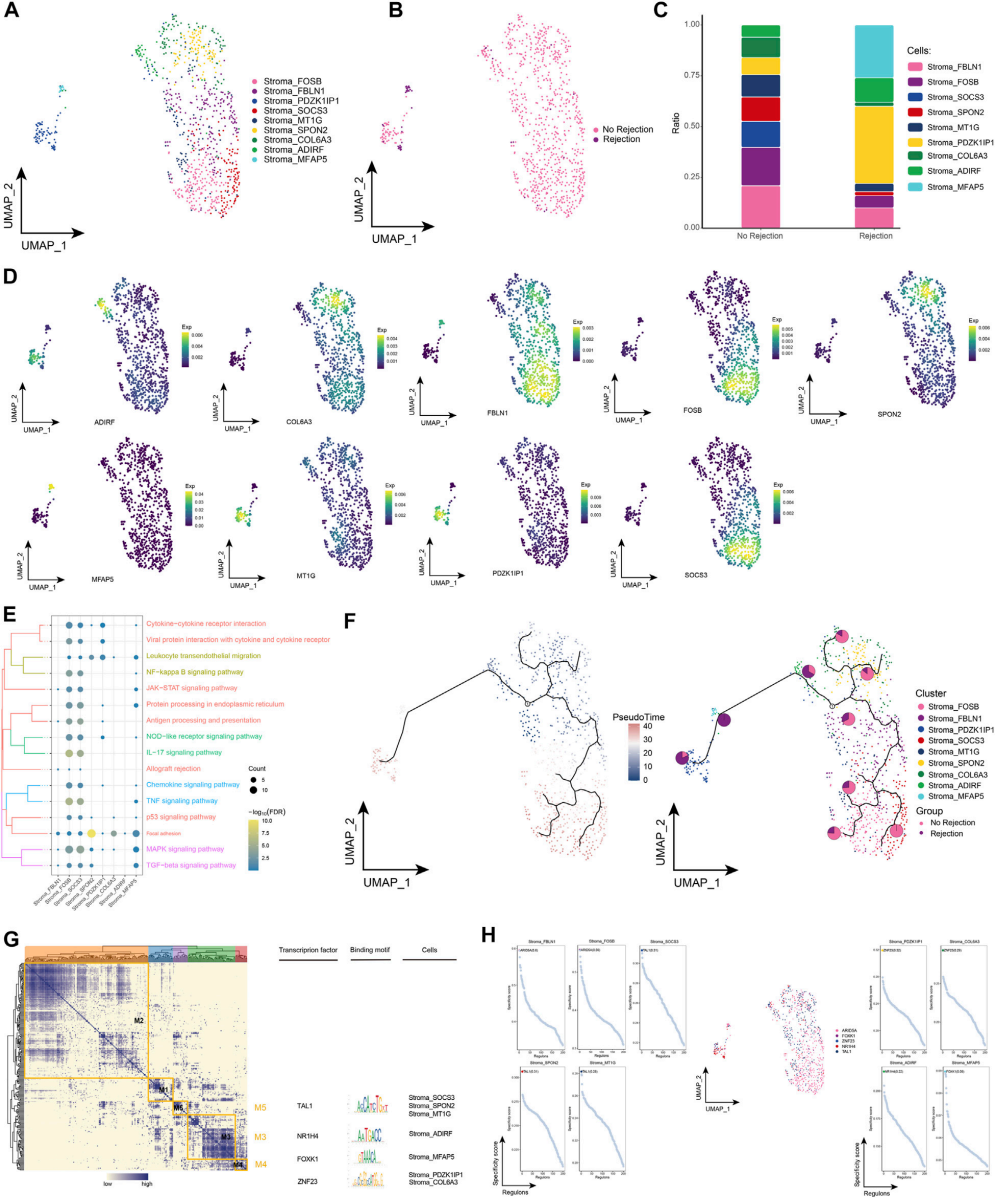
Stroma cell subpopulations in renal transplant patients **(A)**. Single-cell atlas demonstrating into Stroma cell subpopulations. **(B)** Single-cell atlas demonstrating Stroma cell subpopulations in renal transplant patients with and without rejection. **(C)** Differences in abundance of Stroma cell subpopulations in renal transplant patients with and without rejection. **(D)** Markers predominantly expressed by Stroma cell subpopulations. **(E)** Biological processes and signaling pathways underlying the significantly increased enrichment of Stroma cell subpopulations in the microenvironment of renal transplant patients. **(F)** Pseudotime developmental trajectory of Stroma cell subpopulations, with pie charts representing the proportion of Stroma cell subpopulations in patients with and without rejection kidney transplantation. **(G)** Co-expression modules of transcription factors in the Stroma cell subpopulations of kidney transplant patients. Left: Identification of regulator modules based on the regulator’s linkage specificity index matrix. Middle: representative transcription factors and their binding patterns in the modules. Right: cell subpopulations in which transcription factors are located. **(H)** Single-cell atlas showing transcription factors regulating Stroma cell subpopulations.

Stromal cells play a key role in pro-inflammatory and pro-fibrotic activity in renal transplantation (Venner et al., 2016; Cippa et al., 2019). By subpopulation analysis, we identified a total of 9 subpopulations of stromal cells and observed the distribution of subpopulations in different patient groups (Figures 6A, B). Among them, we also found that Stromal_PDZK1IP1 and Stromal_MFAP5 had the highest proportion in patients with ABMR compared to patients with a nonrejection cause, and conversely, Stromal_FBLN1 and Stromal_FOSB had a lower proportion (Figure 6C), and marker genes for each stromal cell subpopulation are shown in Figure 6D. Enrichment analysis revealed that these stromal cell subpopulations were significantly enriched for post-transplant rejection-related pathways and pro-inflammatory pathways, and specifically, cell signaling pathways for adhesion were also enriched (Figure 6E). The pseudotime analysis showed that Stromal_PDZK1IP1 and Stromal_MFAP5 were at the end of the developmental trajectory of stromal cells (Figure 6F). In addition, GRN analysis revealed that stromal cell subpopulations were regulated by TFs such as TAL1, NR1H4, and FOXK1, respectively (Figures 6G, H). In conclusion, by identifying subpopulations of stromal cells in renal transplant patients, we explored the role of stromal cell involvement in allograft rejection.

### 3.7 Oxidative stress in single cells in renal transplant rejection

**FIGURE 7:**
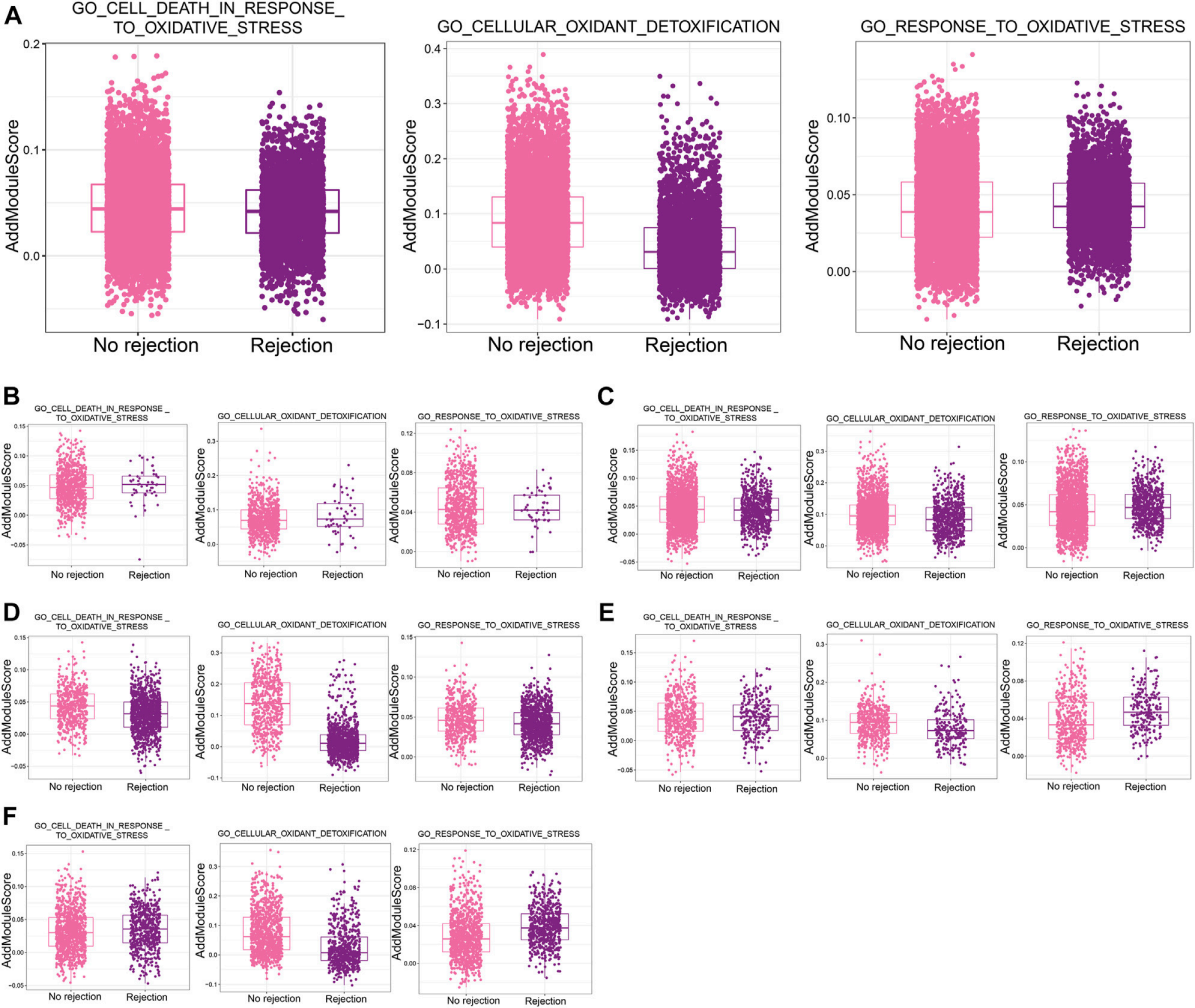
Oxidative stress in single cells in renal transplant rejection **(A)**. Differences in the AddModuleScore of oxidative stress-related biological processes between the non-rejection and rejection groups. **(B–F)** Differences in the AddModuleScore of oxidative stress-related biological processes between the Non-rejection and rejection groups in Stroma cells **(B)**, Ep **(C)**, fibroblasts **(D)**, LOH cells **(E)**, and Macrophages **(F)**.

Calculation of scores for oxidative stress-related biological processes in single-cell samples using AddModuleScore revealed much lower scores for GO_CELLULAR_OXIDANT_DETOXIFICATION in patients with a nonrejection cause than in patients with ABMR (Figure 7A). In addition, stromal cells (Figure 7B), Ep (Figure 7C), fibroblasts (Figure 7D), LOH cells (Figure 7E), and macrophage (Figure 7F) were differentially active in response to oxidative stress, indicating a strong influence by reactive oxygen species in renal transplant rejection.

## 4 Discussion

In this study, we present a comprehensive overview of cell types and subpopulations in kidney biopsy samples from three patients with ABMR and two patients with a nonrejection cause of AKI at single-cell resolution. By assessing the ecological changes and different signaling profiles of subpopulations of different cell types and analyzing the developmental trajectories and transcriptional regulators of different cell subpopulations, our data will help advance our insights into transcriptional differences between cells of different origins in the post-transplant rejection response of kidneys.

First, our analysis identified 11 independent cell types, including a number of renal tubular cell types, epithelial cell types, immune cells, stromal cells, and endothelial cells. Second, each cell type exhibited different pathway characteristics and activity between patients with ABMR and patients with a nonrejection cause of AKI or between each other.

Tissue remodeling is dependent on mesenchymal cells (fibroblasts and myofibroblasts), which is a prominent feature of chronic rejection in allograft kidneys (Grimm et al., 2001). In our study, the increased proportion of fibroblasts in the kidney biopsy tissue of patients with ABMR confirmed that loss of allograft function and the development of interstitial fibrosis in patients with allograft rejection are associated, consistent with previous observations (Toki et al., 2014). The fibroblast subpopulations Fibroblasts_PLVAP and Fibroblasts_CXCL9, plasma vesicle-associated protein (PLVAP) genes identified in ABMR patients are strongly associated with chronic allogeneic rejection and are associated with TG grading and proteinuria (Yamamoto et al., 2007). In contrast, earlier studies have demonstrated that urinary CXCL9 and CXCL10 levels may be predictive of T-cell-mediated rejection in the early post-transplant period and that measurement of urinary CXCL9 and CXCL10 levels may provide an additional tool for the diagnosis of rejection (Ciftci et al., 2019). In addition, it has been recognized that inflammation is a key process leading to progressive renal fibrosis (Meng et al., 2014). In an enrichment analysis of fibroblast subpopulations, these subpopulations were found to be significantly involved in a number of inflammation-related pathways. Although fibroblasts may stimulate renal fibrosis in an indirect manner through the production of pro-inflammatory and pro-fibroblastic cytokines and growth factors (Nikolic-Paterson et al., 2011), a direct link between fibroblasts and myofibroblast accumulation during chronic allogeneic rejection remains undefined. In addition, TGF-β signaling pathway, epithelial-to-mesenchymal transition (EMT) and angiogenesis-related pathways are enriched by fibroblast subpopulations and TGF-β 1 is an important mediator of renal fibrosis after renal transplantation (Campistol et al., 2001). In the context of renal fibrosis, it is necessary for the transition of renal tubular epithelial and endothelial cells to mesenchymal cells (Sato et al., 2003; Yeh et al., 2010; Zhou et al., 2010). Exploring answers to the question of fibroblast subpopulations in allograft kidney biopsies in patients with ABMR and patients with a nonrejection cause of AKI has prompted research into the molecular clues that lead to greater recruitment of fibroblasts to kidneys with rejection, which may have enormous therapeutic implications.

ABMR is commonly associated with glomerular and interstitial inflammation (Magil and Tinckam, 2003; Sund et al., 2004). Activated resident cells, particularly renal tubular epithelial cells, represent a key feature of acute graft rejection and any inflammatory response in natural and transplanted kidneys (Woltman et al., 2000). Several recent evidences suggest that IL-17 can regulate the innate immune response and, to some extent, the adaptive immune response (Ishigame et al., 2009; Sutton et al., 2009). Our study further confirms that renal tubular epithelial cells may serve as immunomodulatory cells in renal allograft rejection.

There is evidence that intra-graft macrophages are associated with poor renal graft outcomes in human and animal models (Matheson et al., 2005; Toki et al., 2014; Ikezumi et al., 2015). In our study, we identified functional heterogeneity among macrophages. Macrophages can promote injury and repair of renal allografts depending on the underlying injury and the effectiveness of immunosuppressive therapy. A subpopulation of macrophages positive for FCN1 was identified in patients with ABMR, FCN1 is involved in the pathogenesis of autoimmune diseases and targeting FCN1 is a promising strategy for the treatment of these diseases (Katayama et al., 2019), however, no studies have yet confirmed the role of FCN1 in renal transplant rejection. In renal transplant rejection, pro-inflammatory macrophages contribute to graft loss and are characterized by the production of pro-inflammatory cytokines and chemokines (IL-1β, MCP-1 and TNF-α) as well as reactive oxygen species production (Qi et al., 2008; Salehi and Reed, 2015), which is consistent with our findings, which found that macrophages appear to be involved in a number of pathways including Chemokine signaling pathway, TNF signaling pathway and Viral protein interaction with cytokine and cytokine receptor in pro-inflammatory processes. In addition, one study found that macrophages can be converted into collagen-producing myofibroblasts, providing a novel mechanism for the direct involvement of macrophages in interstitial fibrosis during chronic renal allogeneic rejection (Wang et al., 2017). This led us to think about the link between macrophages and fibroblasts.

Oxidative stress is a major mediator of adverse outcomes throughout the transplantation process. Transplanted kidneys are susceptible to oxidative stress-mediated injury due to pre- and post-transplant conditions, leading to reperfusion injury or an imbalance between oxidants and antioxidants. In addition to adversely affecting allografts, oxidative stress and its persistent partners, inflammation can lead to cardiovascular disease, cancer, metabolic syndrome and other disorders in transplant recipients (Nafar et al., 2011). We observed significant oxidative stress in patients with rejection after renal transplantation in the present study, and oxidative stress may be associated with various acute injuries that occur after transplantation. However, the study of oxidative stress within single cells in the transplantation population remains elusive and needs to be explored in the future.

In conclusion, we have demonstrated that despite the apparent complexity and heterogeneity of rejection after kidney transplantation, we envision a future in which kidney biopsies subjected to scRNA-seq could be used as part of a molecular diagnostic test. This study reveals hidden transcriptional heterogeneity in population-average measures by analysing scRNA-seq data from kidney biopsies of patients with ABMR and patients with a non-rejection cause of AKI, providing ideas for identifying new targets for personalised therapeutic approaches. However, our analysis has several limitations. First, the sample size was small, and more scRNA-seq data from post-transplant kidney biopsy tissues should be collected for analysis and exploration in the future. Second, this study lacks the analysis of specific cell/gene expression more or less related to specific clinical events, and the single-cell profile behind human kidney transplant rejection obtained in this study can only serve as a reference for subsequent specific cellular/molecular studies, which require further analysis and validation. Finally, this study investigated rejection after kidney transplantation at the single-cell level by bioinformatics techniques, which should be validated by cellular experiments in the future.

## Data Availability

The original contributions presented in the study are included in the article/Supplementary Material, further inquiries can be directed to the corresponding authors.

## Supplementary material

SUPPLEMENTARY FIGURE S1 Quality control number of double cells, dead cells and mitochondrial genes before **(A)** and after **(B)** quality control.

## Abbreviations

scRNA-seq, single-cell RNA sequencing; AKI, acute kidney injury; ABMR, antibody-mediated rejection; GEO, gene expression omnibus; DSA, donor-specific antibodies; UMI, unique molecular identifier; UMAP, uniform manifold approximation and projection; logFC, log fold change; BP, biological processe; GO, gene ontology; KEGG, kyoto encyclopedia of genes and genomes; GRN, gene regulatory network; En, endothelial; Ep, epithelial; Mac, macrophage; LOH, loop of henle; TF, transcription factor; EMT, epithelial-to-mesenchymal transition.

## References

[B1] Augustine J. (2018). Kidney transplant: New opportunities and challenges. Cleve Clin. J. Med. 85 (2), 138–144. 10.3949/ccjm.85gr.18001 29425089

[B2] Becht E. McInnes L. Healy J. Dutertre C. A. Kwok I. W. H. Ng L. G. (2018). Dimensionality reduction for visualizing single-cell data using UMAP. Nat. Biotechnol. 37, 38–44. 10.1038/nbt.4314 30531897

[B3] Butler A. Hoffman P. Smibert P. Papalexi E. Satija R. (2018). Integrating single-cell transcriptomic data across different conditions, technologies, and species. Nat. Biotechnol. 36 (5), 411–420. 10.1038/nbt.4096 29608179 PMC6700744

[B4] Campistol J. M. Inigo P. Larios S. Bescos M. Oppenheimer F. (2001). Role of transforming growth factor-beta1 in the progression of chronic allograft nephropathy. Nephrol. Dial. Transpl. 16 (Suppl. 1), 114–116. 10.1093/ndt/16.suppl_1.114 11369837

[B5] Ciftci H. S. Tefik T. Savran M. K. Demir E. Caliskan Y. Ogret Y. D. (2019). Urinary CXCL9 and CXCL10 levels and acute renal graft rejection. Int. J. Organ Transpl. Med. 10 (2), 53–63. PMC6604756 31285802

[B6] Cippa P. E. Liu J. Sun B. Kumar S. Naesens M. McMahon A. P. (2019). A late B lymphocyte action in dysfunctional tissue repair following kidney injury and transplantation. Nat. Commun. 10 (1), 1157. 10.1038/s41467-019-09092-2 30858375 PMC6411919

[B7] Cooper J. E. (2020). Evaluation and treatment of acute rejection in kidney allografts. Clin. J. Am. Soc. Nephrol. 15 (3), 430–438. 10.2215/CJN.11991019 32066593 PMC7057293

[B8] Der E. Ranabothu S. Suryawanshi H. Akat K. M. Clancy R. Morozov P. (2017). Single cell RNA sequencing to dissect the molecular heterogeneity in lupus nephritis. JCI Insight 2 (9), e93009. 10.1172/jci.insight.93009 28469080 PMC5414553

[B9] Eskandary F. Regele H. Baumann L. Bond G. Kozakowski N. Wahrmann M. (2018). A randomized trial of bortezomib in late antibody-mediated kidney transplant rejection. J. Am. Soc. Nephrol. 29 (2), 591–605. 10.1681/ASN.2017070818 29242250 PMC5791086

[B10] Grimm P. C. Nickerson P. Jeffery J. Savani R. C. Gough J. McKenna R. M. (2001). Neointimal and tubulointerstitial infiltration by recipient mesenchymal cells in chronic renal-allograft rejection. N. Engl. J. Med. 345 (2), 93–97. 10.1056/NEJM200107123450203 11450677

[B11] Ikezumi Y. Suzuki T. Yamada T. Hasegawa H. Kaneko U. Hara M. (2015). Alternatively activated macrophages in the pathogenesis of chronic kidney allograft injury. Pediatr. Nephrol. 30 (6), 1007–1017. 10.1007/s00467-014-3023-0 25487670

[B12] Ishigame H. Kakuta S. Nagai T. Kadoki M. Nambu A. Komiyama Y. (2009). Differential roles of interleukin-17A and -17F in host defense against mucoepithelial bacterial infection and allergic responses. Immunity 30 (1), 108–119. 10.1016/j.immuni.2008.11.009 19144317

[B13] Katayama M. Ota K. Nagi-Miura N. Ohno N. Yabuta N. Nojima H. (2019). Ficolin-1 is a promising therapeutic target for autoimmune diseases. Int. Immunol. 31 (1), 23–32. 10.1093/intimm/dxy056 30169661 PMC6364620

[B14] Li J. Yang K. Y. Tam R. C. Y. Chan V. W. Lan H. Y. Hori S. (2019). Regulatory T-cells regulate neonatal heart regeneration by potentiating cardiomyocyte proliferation in a paracrine manner. Theranostics 9 (15), 4324–4341. 10.7150/thno.32734 31285764 PMC6599663

[B15] Liao J. Yu Z. Chen Y. Bao M. Zou C. Zhang H. (2020). Single-cell RNA sequencing of human kidney. Sci. Data 7 (1), 4. 10.1038/s41597-019-0351-8 31896769 PMC6940381

[B16] Lim M. A. Kohli J. Bloom R. D. (2017). Immunosuppression for kidney transplantation: Where are we now and where are we going? Transpl. Rev. Orl. 31 (1), 10–17. 10.1016/j.trre.2016.10.006 28340885

[B17] Magil A. B. Tinckam K. (2003). Monocytes and peritubular capillary C4d deposition in acute renal allograft rejection. Kidney Int. 63 (5), 1888–1893. 10.1046/j.1523-1755.2003.00921.x 12675868

[B18] Malone A. F. Wu H. Fronick C. Fulton R. Gaut J. P. Humphreys B. D. (2020). Harnessing expressed single nucleotide variation and single cell RNA sequencing to define immune cell chimerism in the rejecting kidney transplant. J. Am. Soc. Nephrol. 31 (9), 1977–1986. 10.1681/ASN.2020030326 32669324 PMC7461682

[B19] Matheson P. J. Dittmer I. D. Beaumont B. W. Merrilees M. J. Pilmore H. L. (2005). The macrophage is the predominant inflammatory cell in renal allograft intimal arteritis. Transplantation 79 (12), 1658–1662. 10.1097/01.tp.0000167099.51275.ec 15973166

[B20] Meng X. M. Nikolic-Paterson D. J. Lan H. Y. (2014). Inflammatory processes in renal fibrosis. Nat. Rev. Nephrol. 10 (9), 493–503. 10.1038/nrneph.2014.114 24981817

[B21] Nafar M. Sahraei Z. Salamzadeh J. Samavat S. Vaziri N. D. (2011). Oxidative stress in kidney transplantation: Causes, consequences, and potential treatment. Iran. J. Kidney Dis. 5 (6), 357–372. 22057066

[B22] Nikolic-Paterson D. J. Wang S. Lan H. Y. (2011). Macrophages promote renal fibrosis through direct and indirect mechanisms. Kidney Int. Suppl. 4(1), 34–38. 10.1038/kisup.2014.7 PMC4536961 26312148

[B23] Qi F. Adair A. Ferenbach D. Vass D. G. Mylonas K. J. Kipari T. (2008). Depletion of cells of monocyte lineage prevents loss of renal microvasculature in murine kidney transplantation. Transplantation 86 (9), 1267–1274. 10.1097/TP.0b013e318188d433 19005409

[B24] Rogers N. M. Zhang Z. J. Wang J. J. Thomson A. W. Isenberg J. S. (2016). CD47 regulates renal tubular epithelial cell self-renewal and proliferation following renal ischemia reperfusion. Kidney Int. 90 (2), 334–347. 10.1016/j.kint.2016.03.034 27259369

[B25] Salehi S. Reed E. F. (2015). The divergent roles of macrophages in solid organ transplantation. Curr. Opin. Organ Transpl. 20 (4), 446–453. 10.1097/MOT.0000000000000209 PMC4520531 26154913

[B26] Salehi S. Shahi A. Afzali S. Keshtkar A. A. Farashi Bonab S. Soleymanian T. (2020). Transitional immature regulatory B cells and regulatory cytokines can discriminate chronic antibody-mediated rejection from stable graft function. Int. Immunopharmacol. 86, 106750. 10.1016/j.intimp.2020.106750 32652501

[B27] Saliba A. E. Westermann A. J. Gorski S. A. Vogel J. (2014). Single-cell RNA-seq: Advances and future challenges. Nucleic Acids Res. 42 (14), 8845–8860. 10.1093/nar/gku555 25053837 PMC4132710

[B28] Sato M. Muragaki Y. Saika S. Roberts A. B. Ooshima A. (2003). Targeted disruption of TGF-beta1/Smad3 signaling protects against renal tubulointerstitial fibrosis induced by unilateral ureteral obstruction. J. Clin. Investig. 112 (10), 1486–1494. 10.1172/JCI19270 14617750 PMC259132

[B29] Sellares J. de Freitas D. G. Mengel M. Reeve J. Einecke G. Sis B. (2012). Understanding the causes of kidney transplant failure: The dominant role of antibody-mediated rejection and nonadherence. Am. J. Transpl. 12 (2), 388–399. 10.1111/j.1600-6143.2011.03840.x 22081892

[B30] Stuart T. Butler A. Hoffman P. Hafemeister C. Papalexi E. Mauck W. M. 3rd (2019). Comprehensive integration of single-cell data. Cell. 177 (7), 1888–1902.e21. 10.1016/j.cell.2019.05.031 31178118 PMC6687398

[B31] Sund S. Reisaeter A. V. Scott H. Mollnes T. E. Hovig T. (2004). Glomerular monocyte/macrophage influx correlates strongly with complement activation in 1-week protocol kidney allograft biopsies. Clin. Nephrol. 62 (2), 121–130. 10.5414/cnp62121 15356969

[B32] Sutton C. E. Lalor S. J. Sweeney C. M. Brereton C. F. Lavelle E. C. Mills K. H. (2009). Interleukin-1 and IL-23 induce innate IL-17 production from gammadelta T cells, amplifying Th17 responses and autoimmunity. Immunity 31 (2), 331–341. 10.1016/j.immuni.2009.08.001 19682929

[B33] Tirosh I. Izar B. Prakadan S. M. Wadsworth M. H. 2nd Treacy D. Trombetta J. J. (2016). Dissecting the multicellular ecosystem of metastatic melanoma by single-cell RNA-seq. Science 352 (6282), 189–196. 10.1126/science.aad0501 27124452 PMC4944528

[B34] Toki D. Zhang W. Hor K. L. Liuwantara D. Alexander S. I. Yi Z. (2014). The role of macrophages in the development of human renal allograft fibrosis in the first year after transplantation. Am. J. Transpl. 14 (9), 2126–2136. 10.1111/ajt.12803 25307039

[B35] Trapnell C. Cacchiarelli D. Grimsby J. Pokharel P. Li S. Morse M. (2014). The dynamics and regulators of cell fate decisions are revealed by pseudotemporal ordering of single cells. Nat. Biotechnol. 32 (4), 381–386. 10.1038/nbt.2859 24658644 PMC4122333

[B36] Valenzuela N. M. Reed E. F. (2017). Antibody-mediated rejection across solid organ transplants: Manifestations, mechanisms, and therapies. J. Clin. Investig. 127 (7), 2492–2504. 10.1172/JCI90597 28604384 PMC5490786

[B37] Van de Sande B. Flerin C. Davie K. De Waegeneer M. Hulselmans G. Aibar S. (2020). A scalable SCENIC workflow for single-cell gene regulatory network analysis. Nat. Protoc. 15 (7), 2247–2276. 10.1038/s41596-020-0336-2 32561888

[B38] Venner J. M. Famulski K. S. Reeve J. Chang J. Halloran P. F. (2016). Relationships among injury, fibrosis, and time in human kidney transplants. JCI Insight 1 (1), e85323. 10.1172/jci.insight.85323 27699214 PMC5033890

[B39] Wang L. Rondaan C. de Joode A. A. E. Raveling-Eelsing E. Bos N. A. Westra J. (2021). Changes in T and B cell subsets in end stage renal disease patients before and after kidney transplantation. Immun. Ageing 18 (1), 43. 10.1186/s12979-021-00254-9 34749733 PMC8574047

[B40] Wang Y. Y. Jiang H. Pan J. Huang X. R. Wang Y. C. Huang H. F. (2017). Macrophage-to-Myofibroblast transition contributes to interstitial fibrosis in chronic renal allograft injury. J. Am. Soc. Nephrol. 28 (7), 2053–2067. 10.1681/ASN.2016050573 28209809 PMC5491278

[B41] Wekerle T. Segev D. Lechler R. Oberbauer R. (2017). Strategies for long-term preservation of kidney graft function. Lancet 389 (10084), 2152–2162. 10.1016/S0140-6736(17)31283-7 28561006

[B42] Woltman A. M. Deh S. Boonstra J. G. Gobin S. J. P. Daha M. R. Kooten C. V. (2000). Interleukin-17 and CD40-ligand synergistically enhance cytokine and chemokine production by renal epithelial cells. J. Am. Soc. Nephrol. 11 (11), 2044–2055. 10.1681/ASN.V11112044 11053480

[B43] Wu H. Malone A. F. Donnelly E. L. Kirita Y. Uchimura K. Ramakrishnan S. M. (2018). Single-cell transcriptomics of a human kidney allograft biopsy specimen defines a diverse inflammatory response. J. Am. Soc. Nephrol. 29 (8), 2069–2080. 10.1681/ASN.2018020125 29980650 PMC6065085

[B44] Yamamoto I. Horita S. Takahashi T. Tanabe K. Fuchinoue S. Teraoka S. (2007). Glomerular expression of plasmalemmal vesicle-associated protein-1 in patients with transplant glomerulopathy. Am. J. Transpl. 7 (8), 1954–1960. 10.1111/j.1600-6143.2007.01876.x 17617859

[B45] Yeh Y. C. Wei W. C. Wang Y. K. Lin S. C. Sung J. M. Tang M. J. (2010). Transforming growth factor-{beta}1 induces Smad3-dependent {beta}1 integrin gene expression in epithelial-to-mesenchymal transition during chronic tubulointerstitial fibrosis. Am. J. Pathol. 177 (4), 1743–1754. 10.2353/ajpath.2010.091183 20709799 PMC2947271

[B46] Yu G. Wang L. G. Han Y. He Q. Y. (2012). clusterProfiler: an R package for comparing biological themes among gene clusters. OMICS 16 (5), 284–287. 10.1089/omi.2011.0118 22455463 PMC3339379

[B47] Zhao Y. Zhang P. Ge W. Feng Y. Li L. Sun Z. (2020). Alginate oligosaccharides improve germ cell development and testicular microenvironment to rescue busulfan disrupted spermatogenesis. Theranostics 10 (7), 3308–3324. 10.7150/thno.43189 32194870 PMC7053202

[B48] Zhou L. Fu P. Huang X. R. Liu F. Chung A. C. Lai K. N. (2010). Mechanism of chronic aristolochic acid nephropathy: Role of Smad3. Am. J. Physiol. Ren. Physiol. 298 (4), F1006–F1017. 10.1152/ajprenal.00675.2009 20089673

