# Supplementary figures and images for: Immune landscape in rejection of renal transplantation revealed by high-throughput single-cell RNA sequencing
Source: Front Cell Dev Biol. 2023 Jul 20;11:1208566. doi: 10.3389/fcell.2023.1208566 (PMC10397399; doi:10.3389/fcell.2023.1208566)

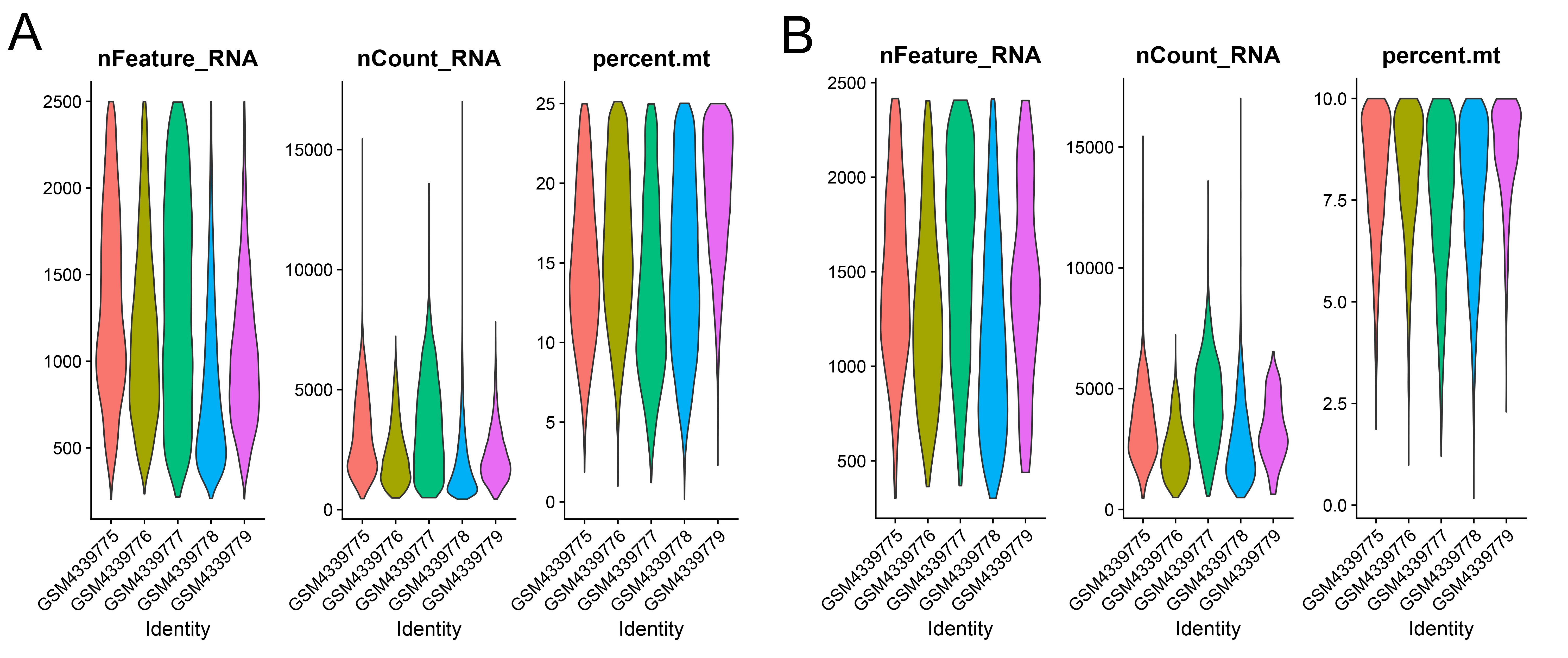

Supplement: Supplementary file 2 [file Image1.TIF]
